# Major contributors to motorcycle accidents in Busia County, Kenya

**DOI:** 10.11604/pamj.2025.51.10.41577

**Published:** 2025-05-12

**Authors:** Olipher Makwaga, Tom Mokaya, Priscah Otambo, Matilu Mwau, Ferdinard Adungo

**Affiliations:** 1 Kenya Medical Research Institute, Department of Biomedical Research, Busia, Kenya

**Keywords:** Factors, motorcycle riders, accidents

## Abstract

**Introduction:**

in Busia, motorcycles are a significant contributor to the number of road traffic injuries. Despite the impact they have on the healthcare system, motorcycle accidents have not received much attention due to a lack of local data and inadequate public policy responses in the country. Therefore, this study aimed to identify the risk factors that predict motorcycle accidents.

**Methods:**

this study utilized a cross-sectional mixed-method approach involving interviews with 423 motorcycle riders to explore factors associated with motorcycle accidents. Additionally, seven focus group discussions were conducted to gather more in-depth information. Data analysis was carried out using multiple logistic regression.

**Results:**

out of the 423 riders interviewed, 243 (57.5%) had been involved in an accident. The likelihood of being in an accident was higher for riders who had consumed alcohol (OR=1.8, 95% CI: 1.2-2.7, p=0.004) and for those not using reflector jackets (OR=2.5, 95% CI: 1.4-4.4, p=0.002). Riders who carried only one passenger at a time were at lower risk (OR=0.6, 95% CI: 0.3-1.0, p=0.037) compared to those carrying multiple passengers. Overtaking from both sides increased the risk of accidents compared to overtaking from the right side (OR=2.1, 95% CI 1.3-3.5, p=0.003). Additionally, not wearing helmets and a lack of driving training were found to be associated with accidents (chi-square, p < 0.05). Qualitative analysis revealed similar factors as predictors of motorcycle accidents as the quantitative findings.

**Conclusion:**

the results indicated several major contributors to motorcycle accidents. The study shows that riders do not adhere to traffic rules. Legal action is needed for those who do not comply with traffic rules and regulations.

## Introduction

Globally, more than 50 million people are injured, and approximately 1.2 million lives are lost annually due to road accidents [1,2]. Road traffic injury (RTI) fatalities are highest in developing countries [3-5], with motorcycle accidents responsible for a significant portion of these fatal incidents [6]. Reported prevalence of injuries ranges from 21-58% in Uganda [7], 55.1% in Ethiopia [8], 12.8 - 60% in previous studies in Nigeria, and around 39.4% in previous studies in Kenya [9-11]. The number of commercial riders has significantly increased in recent years, leading to a risk of dying from a motorcycle accident that is estimated to be 20 times higher than from a motor vehicle accident [6,12]. Motorcycle accidents are a significant yet overlooked emerging public health problem and a leading cause of death among the economically productive age group of 20 to 35 years. This issue primarily affects motorcyclists, passengers, and pedestrians. Many developing nations are experiencing a growing problem of rising fatalities and disabilities due to motorcycle-related injuries, with vulnerable groups bearing the brunt of these consequences [12-15].

The exposure of motorcycle riders on the road results in the highest public health burden when measured in terms of disability-adjusted life years lost. Several factors contribute to motorcycle accidents, including environmental conditions, human error, and faulty motorcycles [16,17]. Motorcyclists are often prone to over speeding and overloading their bikes in pursuit of quick gains. The recklessness, lack of discipline, and disregard for other road users exhibited by these mainly young motorcyclists are major contributors to road-related injuries. A large percentage of motorcyclists do not wear any protective gear, thus increasing their risks of sustaining severe head injuries [12,14,18,19].

Kenya has experienced a significant increase in the number of motorcycles entering the transport sector since 2009. This surge has led to serious road traffic challenges and safety issues in most urban centers, requiring urgent intervention from both county and national governments [18]. Commercial transport using motorcycles commonly known as “boda boda” in East Africa [18] and “okada” in Nigeria [12,20], has become increasingly popular in both rural and urban areas in Busia. For instance, motorcycle transportation services are often quick, especially for short distances in cities and towns. They are efficient in alleviating traffic congestion in urban areas and are available throughout the day and night [13].

The transport industry in Kenya is currently facing its greatest challenge due to the unexpected and rapid expansion of motorcycle use as a commercial mode of transportation. The lack of corresponding expansion and improvement of road infrastructure is particularly [21,22] evident in most towns and urban areas in Kenya, where roads are poor and underdeveloped. Boda boda operations in this area are plagued by various issues, including riders and passengers not wearing helmets, overloading of passengers, lack of proper driver training and licensing, speeding and reckless driving, inadequate regulations and enforcement of traffic rules, and the potential use of alcohol and drugs [12].

The National Transport Safety Authority is currently struggling to enforce safety regulations, specifically for the commercial motorcycle taxi business. In Busia County, for example, the number of injuries related to motorcycles is increasing daily, significantly contributing to reported road traffic injuries. This burden is also draining significant health care resources from the underfunded and poorly prepared county health systems. Despite being a nationwide problem, motorcycle injuries have not received the attention they deserve, partly due to a lack of local data, and public policy responses to this epidemic have been largely ineffective at both the county and national levels. Since the majority of motorcycle injuries are preventable, a clearer understanding of the contributing factors is necessary. Therefore, this study aimed to determine the factors contributing to motorcycle accidents to help mitigate road traffic injuries.

## Methods

**Study area:** the study was conducted in Busia County from January 1^st^ to December 31^st^, 2021. The county is divided into seven sub-counties: Teso-South, Teso-North, Nambale, Butula, Matayos, Funyula, and Budalangi. It is characterized by the cross-border movement of Kenyan and Ugandan pedestrians and motorcycle riders for trading purposes. The county has a population of 893,681 (Kenya National Bureau of Statistics, 2019) [23] and approximately 15,000 motorcycle riders [24].

**Study design:** the study utilized a descriptive cross-sectional design, employing mixed methods of data collection.

**Surveys:** trained research assistants conducted interviews with eligible commercial motorcycle riders at designated stop points or waiting bays during daytime hours (8: 00 am-6: 00 pm). Adult riders who were residents of the respective sub-counties, had been riding for at least three months, and were willing to participate were included in the study. Knowledge, attitude, and practice surveys were conducted to assess general attitudes towards motorcycle-related injuries among riders and awareness of rules and regulations governing commercial motorcycle operation. A total of 423 motorcycle riders were randomly selected for interviews, and 70 for Focused Group Discussion (FGD) at various locations. A multi-stage cluster sampling method was used to select participants to minimize selection bias. The questionnaire included questions on factors influencing or preventing motorcycle accidents, perceptions of traffic regulatory authorities, and enforcement systems. FGD participants were selected from waiting bays, ensuring privacy and confidentiality. Seven FGDs were conducted, one for each sub-county.

**Questionnaire:** the questionnaire was validated by experts and pretested for face-to-face surveys. It was developed based on the study´s objectives, available resources, and format of administration. Clear and concise questions were formulated and evaluated.

**Sample size determination for interview:** for the interview, random sampling was used to ensure representation of various experiences. The sample size was determined using Fisher´s exact test (1998) formula:


$$n=\frac{P\times \left(100-P\right)\times z^{2}}{d^{2}}$$

Where n=minimum sample size, P=0.5 = prevalence of motorcycle accidents, z=1.96 = standard normal deviation, d=0.05 = level of significance.

Substituting appropriately,


$$384=\frac{0.5\times \left(1-0.5\right)\times 1.96^{2}}{0.05^{2}}$$

Minimum sample size was 384. Adjusting for a 10% non-response rate, 423 riders were interviewed.

**Sampling procedures:** Busia County has seven sub-counties and 35 wards. In this study, the sub-counties were the sampling units for the objectives. A sampling frame was created using major motorcycle riders´ stop points and waiting bays for each sub-county. Five waiting bays were randomly selected as sampling units, with 12 riders sampled from each. For FGDs, 10 riders from each of the seven sub-counties, totaling 70 riders.

**Bias:** bias in the study was addressed in a way that ensured representation from all sub-counties and wards of Busia County.

**Data management and analysis plan:** quantitative data collected were double-entered and validated using Microsoft Excel. Descriptive statistics (mean, frequency, and percentage) were utilized to describe the socio-demographic characteristics of the participants and common risk factors associated with motorcycle accidents in the study area. The Chi-square test was employed to assess differences in proportions between variables of interest. Logistic regression was used to analyze the predictors between dependent and independent variables. A simple logistic regression model was used to evaluate significant variables at an alpha level of 0.05 in univariable analysis. The significant variables were then included in a multiple logistic regression model. Data analysis was conducted using STATA version 15.0. Qualitative data from focus group discussions were thematically analyzed.

**Institutional Review Board approval:** this study was approved by the Ethical Review Unit of the Kenya Medical Research Institute under the study number KEMRI/SERU/CIPDCR/008/3639. Permission to conduct a situational analysis of the health facilities was obtained from the county and facility managers.

## Results

**Sociodemographic characteristics:** the total number of participants was 423, with the vast majority being male (421, 99.5%) and only 2 (0.5%) female. The average age of the riders was 32.9 years (S.D. = 8.9, Min - Max = 18 - 67). Most riders had completed primary education (145, 34.3%) or had incomplete primary education (126, 29.8%). A smaller percentage had completed secondary education (70, 16.6%), incomplete secondary education (47, 11.1%), or a college/university education (12, 2.8%). Twenty-two (5.2%) riders reported having no education. The largest ethnic group among the riders was Luhya, accounting for 267 (63.1%) of the total, while other significant ethnic groups included Iteso (124, 29.3%) and Luo (29, 6.9%) (Table 1).

**Table 1:** demographic characteristics of motorcycle riders, recruited from major motorcycle riders’ stop points and waiting bays in Busia County, from 1^st^ January to 31^st^ December 2021 (N=423)

Socio-demographic information	Participants (%)
**Gender**	
Male	421 (99.5)
Female	2 (0.5)
Average age (SD, min-max)	32.9 (8.9, 16-67)
**Level of education**	
College/university	12 (2.8)
Completed secondary	70 (16.6)
Incomplete secondary	47 (11.1)
Complete primary	145 (34.3)
Incomplete secondary	126 (29.8)
None	22 (5.2)
**Ethnic background**	
Luo	29 (6.9)
Luhya	267 (63.1)
Teso	124 (29.3)

**Factors influencing or preventing the occurrence of motorcycle accidents:** out of the 423 participants interviewed, more than half (243, 57.5%) had been involved in an accident. Univariable analysis showed that alcohol intake, reflector usage, wrong overtaking, and overloading (p <0.05) were significant factors influencing or preventing motorcycle accidents. Riders who had ever consumed alcohol were at a higher risk (OR = 1.8, 95% CI: 1.2-2.7, p=0.004) compared to those who had never consumed alcohol. Similarly, riders who didn´t use reflectors were at a significantly higher risk (OR = 2.5, 95% CI: 1.4-4.4, p=0.002) of being involved in an accident than those who did use reflectors. Riders who carried one passenger at a time were at lower risk (OR = 0.6, 95% CI: 0.3-1.0, p=0.037) of being involved in an accident than those who carried more than one passenger. Riders overtaking from both sides had a higher risk of being involved in an accident than riders overtaking from the right side (OR = 2.1, 95% CI 1.3-3.5, p=0.003).

Additionally, not wearing helmets was associated with causing accidents, and a lack of training/driving license was associated with the occurrence of accidents. The data showed that the majority of riders (301, 71.2%) reported having no driving license, while a minority (116, 27.4%) reported having one. The majority of riders (339, 80.1%) reported wearing helmets, while a minority (84, 19.9%) reported not wearing helmets. Of those wearing helmets, 154 (45.2%) were involved in accidents while 182 (53.7%) were not. For those not wearing helmets, 61 (72.6%) were involved in accidents, while 23 (27.4%) were not (Table 2). Other factors like age, level of education, years of riding experience, and type of motorcycle analyzed in the study were not associated with accidents (p values >0.05) (Table 3).

**Table 2:** factors influencing or preventing the occurrence of motorcycle accidents among the riders recruited from major motorcycle’s stop points and waiting bays in Busia County, from 1^st^ January to 31^st^ December 2021 (univariable analysis) (N=423)

Factor	Ever involved in an accident (n=243)		Odds ratio (95% CI)	p-value
	Yes (%)	No (%)		
Currently smoke tobacco products	11 (4.5)	2 (1.1)	4.1 (0.9-19.0)	0.066
Currently use smokeless tobacco products	2 (0.8)	1 (0.6)	2 (0.2-24.2)	0.586
Ever used smokeless tobacco products daily	3 (1.2)	1 (0.6)	2.2 (0.2-21.3)	0.496
Ever consumed any alcohol	118 (48.6)	61 (34.5)	1.8 (1.2-2.7)	0.004**
Consumed alcohol within the past 12 months	53 (21.8)	19 (10.7)	2.3 (1.3-4.1)	0.004**
**Frequency of having at least one standard alcoholic drink**				
1-2 days per week	13 (5.4)	8 (4.5)	Reference	
1-3 days per month	6 (2.5)	2 (1.1)	1.8 (0.3-11.5)	0.511
3-4 days per week	5 (2.1)	3 (1.7)	1.0 (0.2-5.5)	0.976
5-6 days per week	4 (1.7)	0 (0)	Omitted	
Daily	12 (4.9)	0 (0)	Omitted	
Less than once per month	10 (4.1)	5 (2.8)	1.2 (0.3-4.9)	0.769
Consumed alcohol within the past 30 days	47 (19.3)	17 (9.6)	1.1 (0.2-6.2)	0.909
Ever consumed home brewed alcohol	31 (12.8)	10 (5.7)	1.7 (0.6-4.5)	0.301
**Age of the motorcycle**				
11-15 years	1 (0.4)	4 (2.3)	0.2 (0.0-1.5)	0.116
16-20 years	1 (0.4)	0 (0)	Omitted	
6-10 years	37 (15.2)	31 (17.5)	0.8 (0.5-1.3)	0.445
Less than 5 years	202 (83.1)	138 (78.0)	Reference	
Don’t know	0 (0)	1 (0.6)	Omitted	
**Indicator condition**				
Bad	1 (0.4)	3 (1.7)	0.24 (0.0-2.3)	0.219
Good	241 (99.2)	174 (98.3)	Reference	
**Tires condition**				
Bad	2 (0.8)	1 (0.6)	Reference	
Good	240 (98.8)	176 (99.4)	0.7 (0.1-7.6)	0.755
**Use of reflectors**				
Yes	187 (76.9)	157 (88.7)	Reference	
No	53 (21.8)	18 (10.2)	2.5 (1.4-4.4)	0.002**
Sometimes	3 (1.2)	2 (1.1)	0.5 (0.1-3.3)	0.479
**Side when overtaking**				
Left	162 (66.7)	138 (78.0)	Reference	
Right	10 (4.1)	10 (5.7)	0.9 (0.3-2.1)	0.729
Both	70 (28.8)	28 (15.8)	2.1 (1.3-3.5)	0.003**
**People carry at a go**				
1	38 (15.6)	28 (15.8)	Reference	0.212
2	144 (59.3)	120 (67.8)	0.6 (0.3-1.0)	0.037**
3	58 (23.9)	28 (15.8)	0.7 (0.3-1.3)	0.045**
			**Chi-square value**	**P value**
Wearing of helmet	339 (80.1)	84 (19.9)	10.183	0.006**
Driving license	116 (27.4)	307 (72.6)	25.380	0.000**
Condition of the road				

** : factors contribute to an accident because of their p-value of less than 0.05

**Table 3:** factors not associated with motorcycle accidents among the riders recruited from major motorcycle’s stop points and waiting bays in Busia County, from 1^st^ January to 31^st^ December 2021 (N=423)

	Yes accidents	No accidents	Chi-square value	p-value
**Age in years**				
≤ 25	50	32		
26-35	120	90	12.456	0.132
36-45	54	28		
46-55	13	21		
≥ 56	4	6		
No response	3	2		
**Level of highest education**				
None	15	8		
Incomplete primary	68	58	8.854	0.715
Complete primary	82	61		
Incomplete secondary	27	19		
Complete secondary	46	24		
College/university	5	7		
No response	2	1		
**Years of riding experience**				
≤ 5	100	95		
6-10	105	60	7.030	0.318
11-15	33	20		
≥ 16	4	2		
No response	2	2		
**Type of motorcycle riders are using**			3.674	0.721
Bajaj	158	105		
Boxer	52	43		
Honda	9	7		
Others	24	22		
No responses	0	3		

The factors listed in this table were not associated with motorcycle accidents among the riders in Busia County, Kenya, as indicated by their p-values of more than 0.05

**Results of the focus group discussion:** qualitative data were collected using FGD. A major question was designed for response, and the study participants were selected purposively, with data analysis done thematically. About 70 male participants were included in the current study. Their age ranged from 20 to 55 years old (median age 31; interquartile range 28 years). Many participants had been riding motorbikes for more than 3 years.

**The discussion question:** the discussion question was about the contributors to motorcycle accidents, which riders reported by riders as including riding under the influence of alcohol, poor road conditions, lack of knowledge and experience, pride, carelessness, poor leadership by the organizations they are registered with, and emotional stress from “boda boda” lending companies. One rider from Amukura mentioned, “*Some of us ride bikes when we are drunk, the roads are poor, some riders lack knowledge and experience, and stress from loan companies causes accidents*”. Others mentioned that cane lorries are driven at high speeds without observing traffic rules, with only one headlight at night, often mistaken for “boda boda”, leading to accidents. Riders from Nambale and Butula highlighted this issue, stating that accidents happen at night due to this confusion.

Over speeding by riders was another cause of accidents, with riders from Busia stating that it tarnished their reputation and endangered other road users. The use of a salon car (Probox) used for transporting goods and passengers respectively, was mentioned as a cause of accidents by riders from Busia. They criticized the reckless driving and over-speeding of these vehicles. Participants also cited a lack of road signage, overloading, poor maintenance of the bikes due to low income, and unexpected crossing by farm animals as additional causes of accidents. One rider from Funyula emphasized the need for road signage to reduce accidents, while another from Nambale pointed out the dangers of overloading and carrying bulky items. A rider from Angurai highlighted the impact of poor bike maintenance due to financial constraints, leading to increased accidents. Participants from Port-Victoria also complained about illegal speed bumps and the lack of proper signage, which contributed to accidents.

## Discussion

Less than a decade ago, the World Health Organization ranked road traffic injuries as the ninth leading cause of death worldwide [25]. Current projections suggest that these injuries will rise to the seventh leading cause of death by 2030 unless appropriate actions are taken to address the situation [26]. It was for these reasons that the current study was conducted to determine the factors associated with motorcycle accidents in Busia, one of the 47 counties of Kenya. Motorcycle transportation is a common means of travel in both rural and urban areas, leading to a significant level of morbidity and mortality, often leaving those involved partially or completely disabled [27]. In the present study, over half of the riders surveyed reported being involved in accidents while riding. The major contributing factors are highlighted below.

Multiple factors, including behavior and the utilization of personal protective gear, were associated with motorcycle accidents. Inexperienced riders who had not been trained on how to use the road were the leading cause of accidents. This was closely followed by other factors, namely, not using reflectors, overtaking on the wrong side, consumption of alcohol, not wearing helmets while riding, and overloading. Acquiring knowledge and experience through training provides an effective means of reducing accidents. Training increases the safety of riders by enabling them to acquire skills and learn driving laws in the country.

Riders can also identify and correct bad habits, reducing recklessness while riding. Training also boosts their confidence by teaching riding etiquette [28]. Despite all the benefits of acquiring training on how to ride, the majority of the riders in this study were not trained, indicating that a lack of riding skills was a possible cause of accidents. These findings are consistent with a previous review conducted on factors associated with motorcycle-related road traffic crashes in Africa, although lack of training was not the leading cause of motorcycle accidents [29]. While the study population and site were slightly different, our data also aligns with the findings from a similar study on victims of motorcycle crashes at Kagundo Hospital in Machakos County, which reported that a lack of riding skills acquired from driving schools was among the main contributors to motorcycle accidents [30].

Another factor that contributed to the majority of motorcycle accidents in the current study was also reported in other related past studies [29,31]. Overtaking on the wrong side sends a big signal that the riders in the study were ignorant of the safety measures of the road. Many riders are overtaking from the right side, and a considerable number of them were also reported to have overtaken inappropriately. The number of riders overtaking from both the left sides in the current study is worrying as it is associated with causing motorcycle accidents. As reported in previous studies [31], likewise in this study, riders do not follow the same traffic lane but utilize the space between the moving vehicles. This scenario creates varied traffic patterns, which culminate in a high accident threat. Other previous studies have reported overtaking to contribute to accidents [29]; however, the past studies did not disclose the side from which motorcycle riders were overtaking. The behavior of overtaking from the wrong side, as reported in this study during focused group discussion, was linked to alcohol consumption. In the current study, more than a third of motorcyclists ride under the influence of alcohol. Out of these, more than three-quarters have ever caused an accident and been injured. The scenario in this study that alcohol consumption by riders is associated with accidents is in tandem with some other past studies conducted in Africa [29] and elsewhere [32].

The study established that overloading was common among riders, with one motorcycle meant for one passenger often transporting two to three individuals. Overloading of passengers was also found to lead to accidents in a previous study conducted over a decade ago in rural areas of Kagundo in Machakos County [30]. One important piece of personal protective gear that motorcyclists should utilize is reflector jackets. Motorcycles are common small vehicles used for transportation on public roads, so increasing the visibility and alertness of motorcyclists to other road users is crucial [33]. Despite this advantage, some riders in this study did not use reflector jackets, and those who did sometimes did not use them properly. This habit put the riders in the study at a higher risk for motorcycle crashes. The likelihood of riders not using reflector jackets and being involved in accidents in this study was very high, as indicated by the p-value outcome. Our study´s results align with those of a previous study [29].

Utilization of helmets by riders safeguards them from severe head injuries and fatalities [34-36]. However, the majority of riders in the current study do not wear helmets, exposing themselves to a higher risk of accidents. Riding without a helmet exposes the eyes to direct wind and other flying objects in the air, potentially causing loss and accidents.

The findings contradict those of a previous study in the United States of America [37], possibly due to the small sample size in the previous study and the different populations considered in the two studies. Other factors such as age and education level of participants, the condition and age of motorcycles, the type of motorcycle used and the number of years participants have been riding, which have been reported to contribute to motorcycle accidents elsewhere [8,29], were not found to be associated with accidents in the current study. Additional factors contributing to accidents mentioned during focus group discussions included domestic animals crossing the roads unexpectedly, citizens erecting illegal speed bumps, lack of signage, over speeding, poor road conditions, and ineffective government leadership.

**Limitations of the study:** the study did not include interviewing pillion passengers, which could underestimate the overall data. This is because passengers may provide hidden information that could be crucial. We recommend that in these situations, all information should be gathered from both riders and passengers who have been involved in a motorcycle accident.

## Conclusion

The results indicated several major contributors to motorcycle accidents among riders and pillion passengers in Busia County. The study shows that riders do not adhere to traffic rules and regulations. There is a need for concerned agencies to institute mechanisms to safeguard motorbike users. Poor road conditions, lack of road signage, and illegal bumps were mentioned as causes of accidents during focus group discussions. Therefore, improving the roads, erecting appropriate signage, and installing traffic pumps would reduce injuries and fatalities due to motorcycle accidents.

### What is known about this topic

- Despite the availability of training, most riders are not taught how to safely ride on the road;
- Motorcycle accidents result in a significantly higher number of fatalities compared to other types of road traffic accidents.

### What this study adds

- Our findings reveal specific risk factors such as inadequate rider training, riders not observing safety traffic rules, and alcohol consumption;
- By identifying these key contributors, the findings can help policymakers, local authorities, and law enforcement departments in implementing targeted actions such as improved rider training programs and stricter enforcement of traffic rules;
- Additionally, this research adds to the overall body of knowledge on motorcycle safety in Busia County, Kenya, laying the foundation for future studies and comparative investigations in similar settings.
